# Diagnosis and intervention of severe tricuspid regurgitation secondary to rupture of the chordae tendineae: A case report and literature review

**DOI:** 10.3389/fped.2023.1115052

**Published:** 2023-02-08

**Authors:** Jiaxi Huang, Chaojun Du, Wenbo Zhang, Yaping Mi, Yaping Shan, Huifeng Zhang, Qiqi Shi, Gang Chen

**Affiliations:** ^1^ Department of Pediatric Cardiothoracic Surgery, Children's Hospital of Fudan University, Shanghai, China; ^2^ Department of Pediatric Cardiothoracic Surgery, Anhui Provincial Children's Hospital, Anhui, China

**Keywords:** tricuspid regurgitation, rupture, chordae tendinea, neonates, surgery

## Abstract

Unguarded severe tricuspid regurgitation caused by rupture of papillary muscle or chordae tendineae is rare but fatal in neonates. The experience in the management of these patients is still limited. A newborn presenting severe cyanosis after delivery was diagnosed with severe tricuspid regurgitation secondary to rupture of chordae tendineae by echocardiography (Echo), then treated by surgical reconstruction of chordae/papillary muscle connection without artificial materials. A takeaway lesson from this case is that Echo is an important method to diagnose a rupture of chordae tendineae or papillary muscle and that prompt diagnosis and timely surgery can be life-saving.

## Introduction

Severe tricuspid regurgitation (TR) secondary to rupture of the papillary muscles or chordae tendineae in newborns, a rare but potentially lethal condition, is characterized by refractory hypoxemia with typical echocardiography (Echo) findings, including severe TR, compromised antegrade pulmonary flow, and flailed leaflet of the tricuspid valve (TV) (1, 2). Rapid deterioration of heart failure may cause death without prompt diagnosis and effective intervention. Although unclear, it is reported that many perinatal factors contributed to the rupture of the papillary muscles or chordae tendineae, including perinatal hypoxemia, rhesus isoimmunization, premature closure of the ductus arteriosus *in utero*, maternal connective tissue diseases, thromboembolism, and infection (3, 4). However, the experience in the management of these patients remains limited. In this report, we reported our experience of management of a newborn with severe TR secondary to rupture of the chordae tendineae.

## Case description

**Figure 1:**
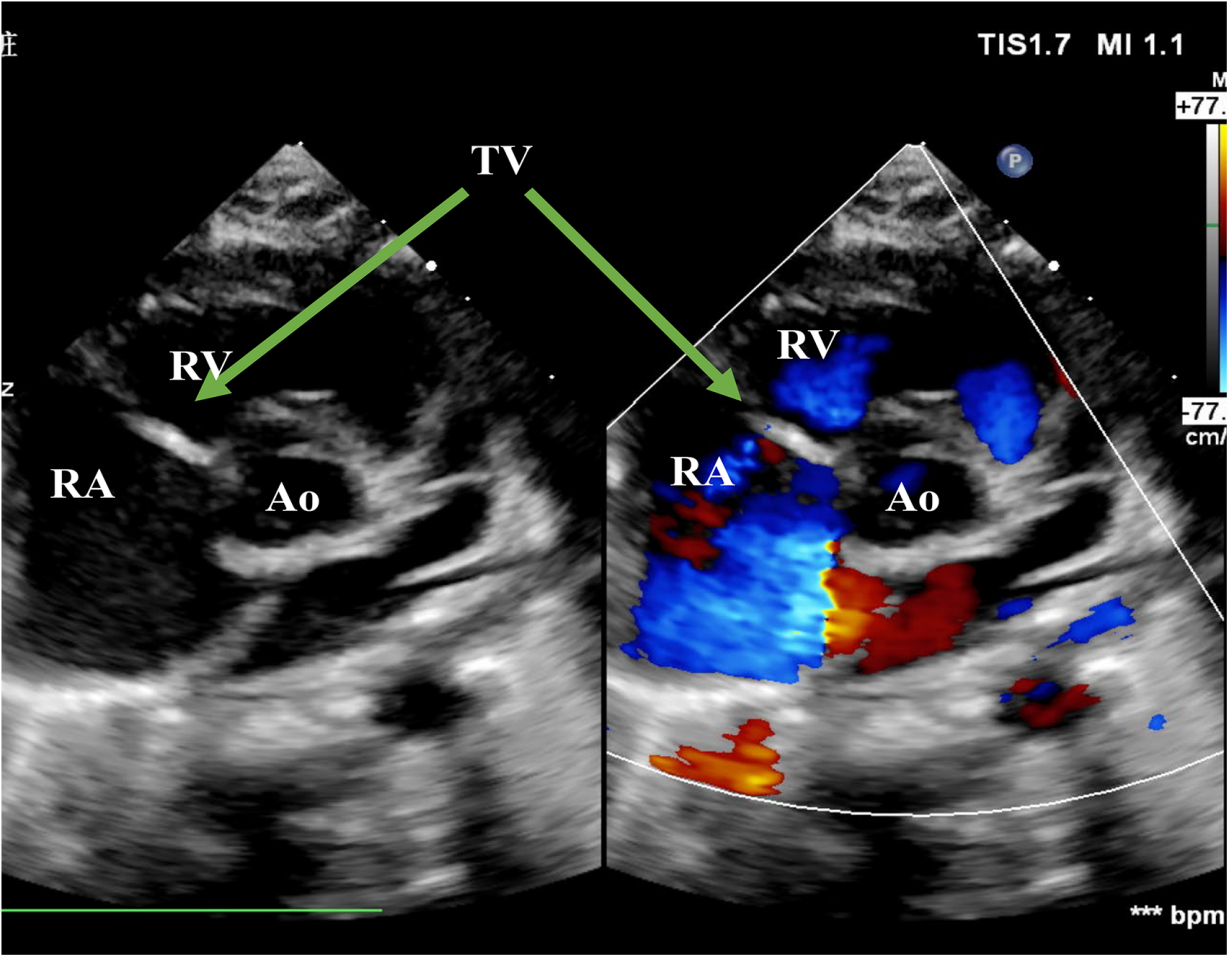
Echo before surgery in the PSAX view. The picture showed severe TR. RA, right atrium; RV, right ventricle; Ao, Aorta; TV, tricuspid valve; PSAX, parasternal short-axis view; TR, tricuspid regurgitation.

**Figure 2:**
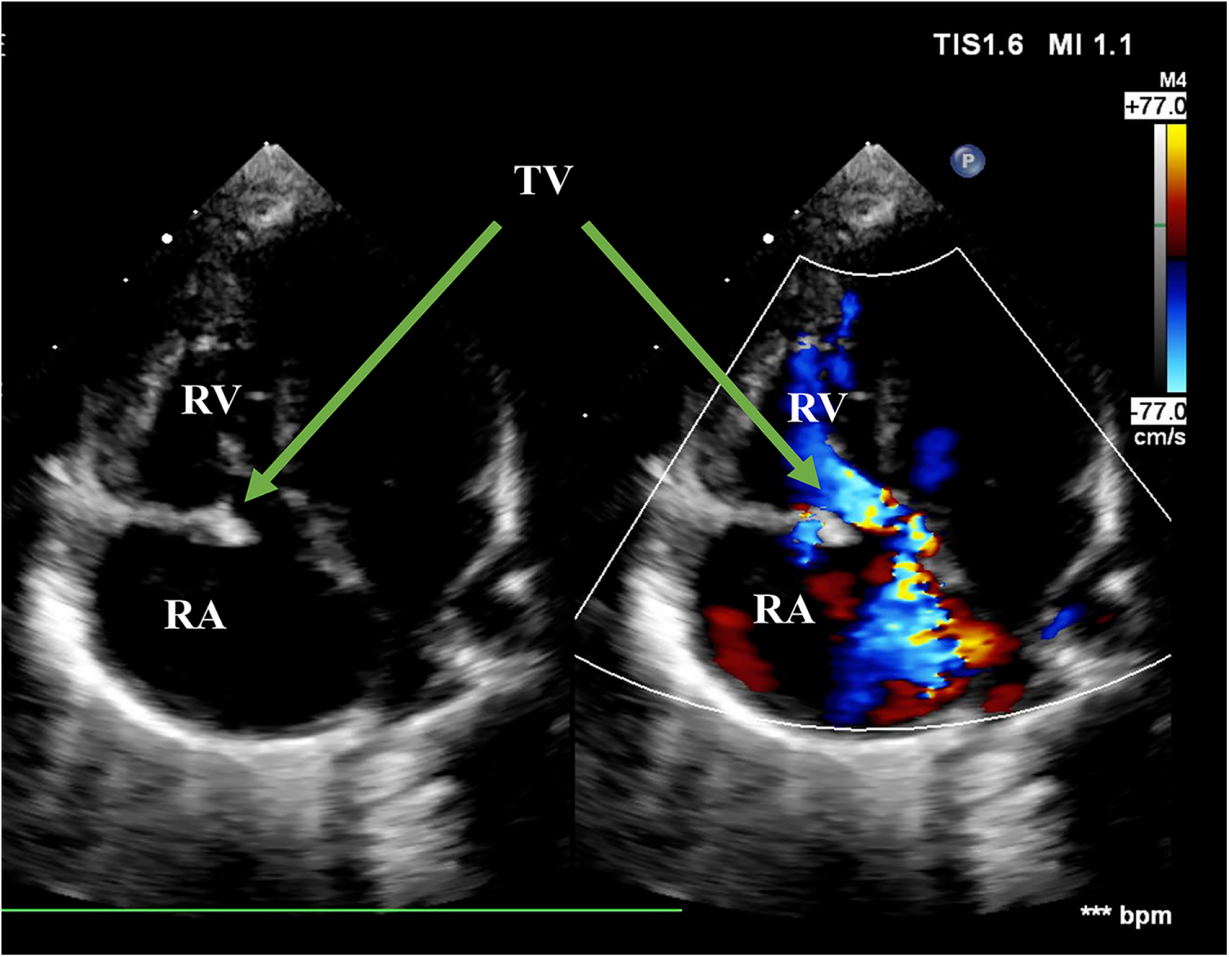
Echo before surgery in the A4C view. The picture showed flailed anterior leaflet prolapsing into the right atrium with a hyperechogenic mass. The green arrows pointed at flailed anterior leaflet of the tricuspid valve. RA, right atrium; RV, right ventricle; TV, tricuspid valve; A4C, apical four-chamber.

A 3,750-g full-term baby born by normal vaginal delivery was found with cyanosis with oxygen saturation of 46.6% and grade III/VI holosystolic murmurs in auscultation on the second day of life. Echocardiogram revealed prolapse of the anterior leaflet, restricted movement of the septal leaflet, and severe tricuspid regurgitation with a peak gradient pressure of 37 mmHg. The antegrade pulmonary flow was compromised with a right-to-left shunt patent foramen ovale (PFO). The diagnosis of the ruptured chordae tendineae was made by the flailed anterior leaflet of the TV with a thickened echogenic tip (Figures 1, 2). Electrocardiogram (ECG) results indicated enlargement of the right atrium and right ventricle. Contrast-enhanced MRI showed mild enlargement of the right atrium and right ventricle, mild pericardial effusion, and a tricuspid regurgitation fraction of 70%. The mother stated a history of normal pregnancy and denied using any drugs during pregnancy including prostaglandin synthetase inhibitors. She denied a family history of congenital heart disease or other hereditary diseases. The baby was stabilized by oxygen supplementation and the administration of milrinone.

**Figure 3:**
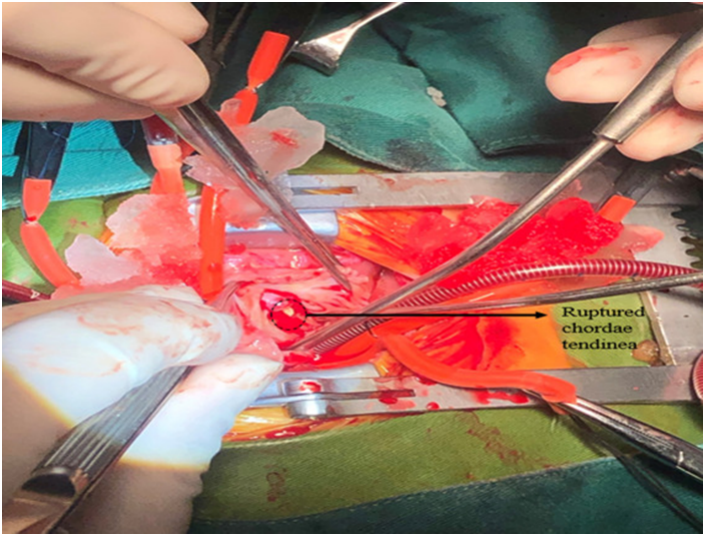
Surgical findings. A ruptured chorda of the anterior leaflet of TV was found, and the other head of papillary muscle was found near the moderator band. No ischemic changes were observed. TV, tricuspid valve.

He was taken up for surgery on day 14 of life. Cardiopulmonary bypass was instituted using aortic and bicava cannulation. The heart was arrested with antegrade blood cardioplegia through the aortic root. A right atriotomy was made to approach the tricuspid valve. After careful exploration, we found that the papillary muscle of the anterior leaflet of the TV was ruptured. Near the moderator band, we found the other end of the ruptured chordae attached to the tip of the anterior papillary muscle (Figure 3). No ischemic change or calcification of the papillary muscles or chordae tendineae was found. The free edge of the fractured chordae was directly sutured to the anterior papillary muscle by a double-head 6-0 prolene suture. Saline was used to distend the ventricle, and a significant reduction of regurgitation was observed. A suture of the commissure of anterior and septal leaflets was done, and then no apparent regurgitation was observed. The 5-mm PFO was closed by a continuous suture. The atriotomy was closed, the aorta was deaired, and the aortic clamp was removed. The heart beats with sinus rhythm and then comes off of cardiopulmonary bypass. The postoperative transesophageal echocardiogram (TEE) revealed trivial regurgitation of the TV after surgery.

**Figure 4:**
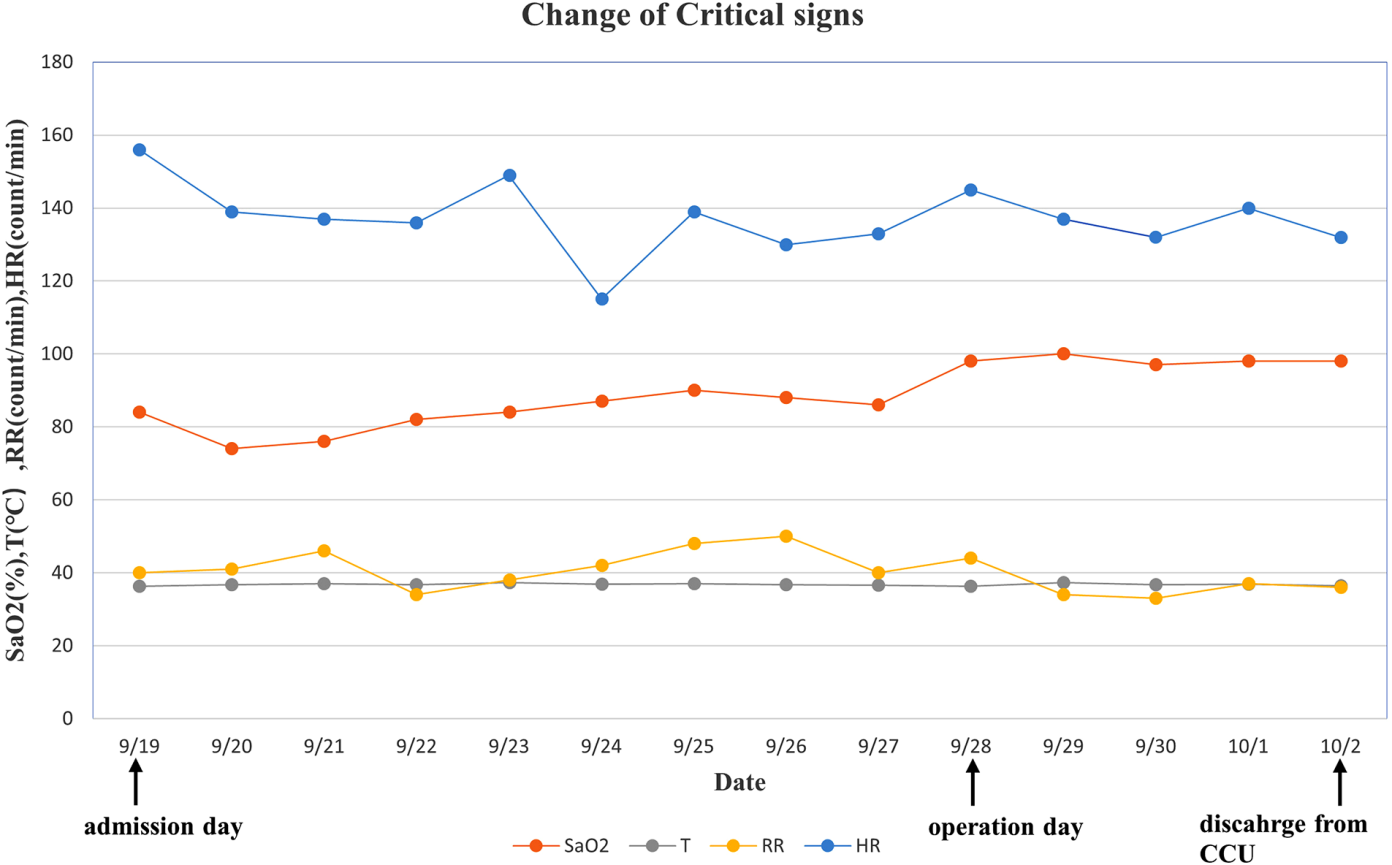
Change of critical signs of the patient. We observed that SaO_2_ increased significantly after surgery. SaO_2_, oxygen saturation; T, temperature; RR, respiratory rate; HR, heart rate.

**Figure 5:**
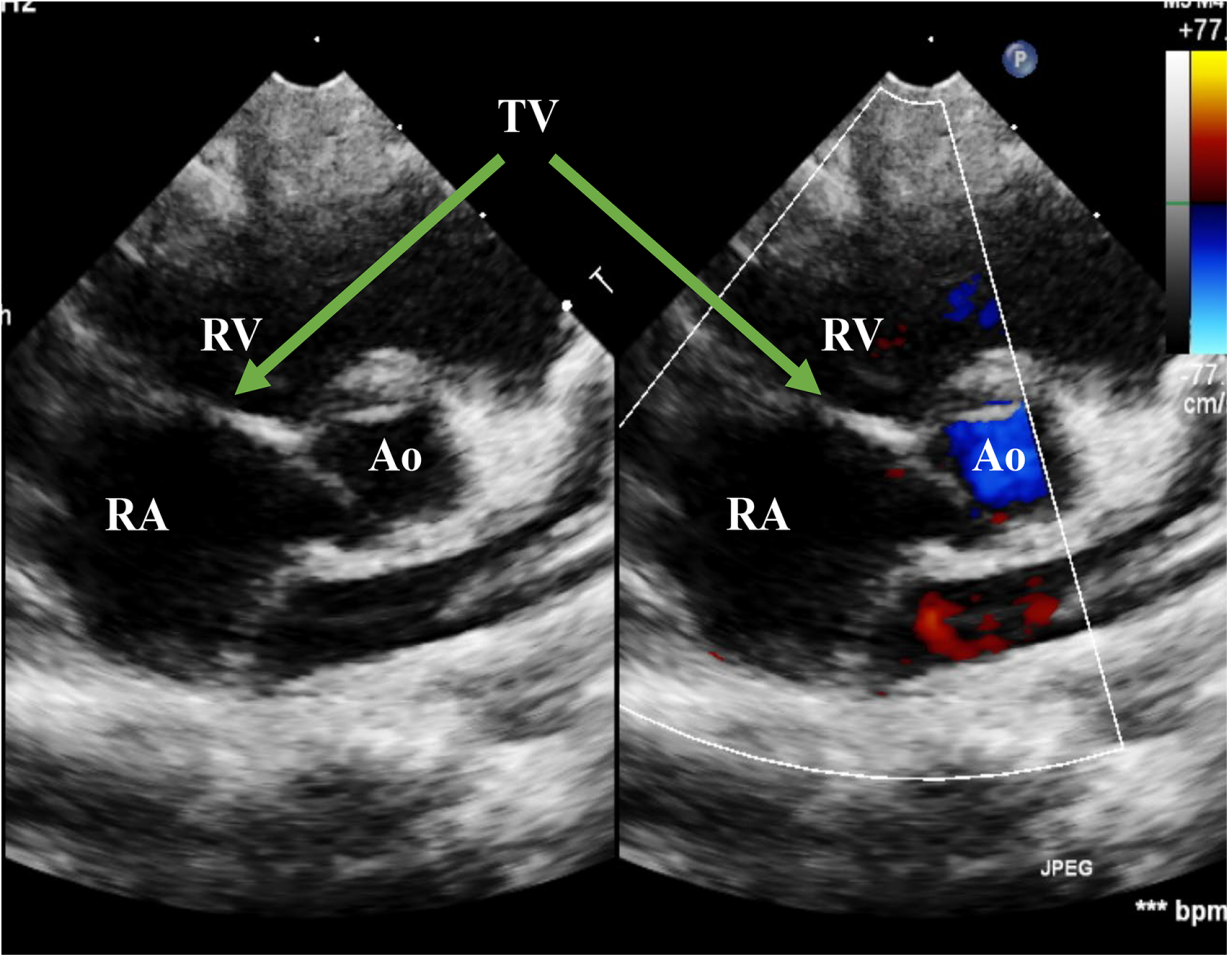
Echo at the latest visit in the PSAX view. The figure showed good coaptation of the TV in the systolic phase. PSAX, parasternal short-axis view; RA, right atrium; RV, right ventricle; Ao, Aorta; TV, tricuspid valve

**Figure 6:**
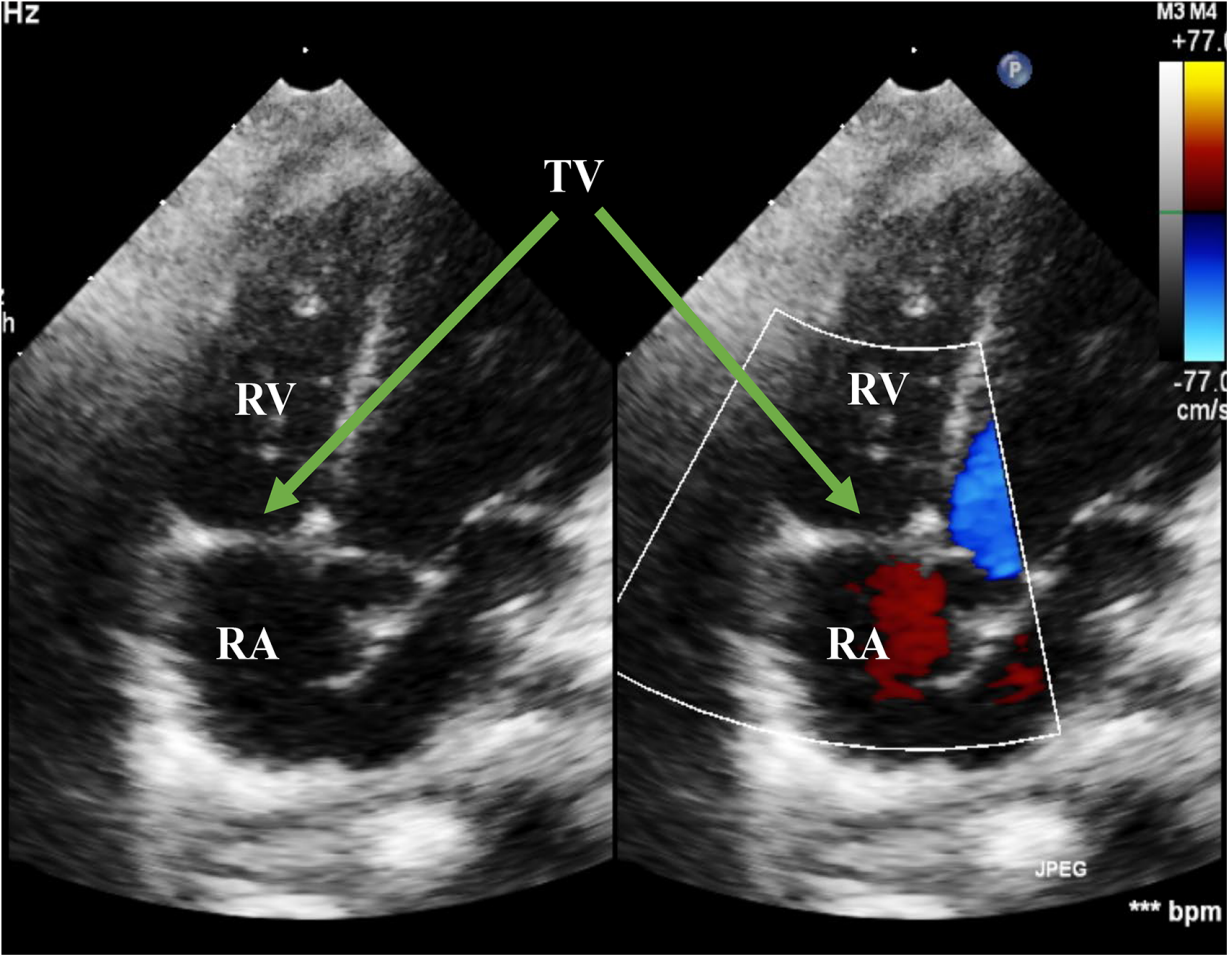
Echo at the latest visit in the A4C view. The figure showed normal movement of the anterior leaflet of the TV in the systolic phase. RA, right atrium; RV, right ventricle; TV, tricuspid valve; A4C, apical four-chamber.

The patient was weaned from mechanical ventilation 12 h after surgery and left the cardiac intense care unit (CICU) on the fourth day after surgery (Figure 4). Inotropic and nitro oxide were not needed after surgery. An Echo before discharge indicated no deterioration of TR, with coordinated movement during the cardiac cycle. ECG before discharge indicated complete right bundle branch block (CRBBB). The critical signs of the patient during the process of management are shown in Figure 3, indicating a quick improvement in oxygen saturation after surgery (Figure 4). The last visit at 4 months old showed normal biventricular function and mild TR (Figures 5, 6).

## Discussion

Isolated tricuspid regurgitation caused by rupture of the papillary muscles or chordae tendineae is rare but fatal in newborns (5, 6). It is important to diagnose promptly through detailed examinations. Patients always present profound cyanosis as severe TR increases the pressure of the right atrium, leading to a large right-to-left shunt, similar to the hemodynamic performance of patients with pulmonary atresia. Echo is the preferred method to confirm a diagnosis. A typical Echo result reveals severe eccentric TR, PFO with right-to-left shunt, and compromised antegrade pulmonary flow at the parasternal short-axis view (PSAX). At the apical four-chamber (A4C) view, there are important findings that differentiate from Ebstein anomaly, including flailed anterior leaflet prolapse to the right atrium during the systolic phase and hyperechogenic papillary muscle lashing with the movement of the leaflet.

Rupture of the papillary muscles or chordae tendineae in neonates can be attributed to ischemic or nonischemic etiologies (3). Ischemic etiologies include perinatal hypoxemia (7), rhesus isoimmunization, premature closure of the ductus arteriosus *in utero*, maternal connective tissue diseases, thromboembolism, and infection (4, 8, 9). Nonischemic etiologies include trauma and infectious endocarditis (3). In fetuses or neonates, the anterior papillary muscle of the tricuspid valve is susceptible to ischemia due to its high oxygen demand and blood supply from the distal extreme of the coronary circulation (10). In addition, overload volume or high pressure of the right ventricle can also cause rupture of the chordae or papillary muscles, as the papillary muscles under high pressure in the systolic phase are more likely to rupture. Premature closure or constriction of ductus arteriosus in fetuses can cause overload volume, high pressure of the right ventricle, and pulmonary hypertension (11). Four articles reported patients with ruptured papillary muscles related to premature constriction of ductus arteriosus, which might be associated with maternal intake of nonsteroidal anti-inflammatory drugs (NSAIDs), structural cardiac lesions such as tetralogy of Fallot, or spontaneous occurrence (11–14). In patients with systemic lupus erythematosus (SLE), the immune reaction within the myocardium extended to the subvalvular apparatus, caused fetal discomfort and hypoxic insult, and finally led to excessive fiber tension, papillary muscle rupture, and critical valve regurgitation (1). In our case, there was not any evidence of perinatal asphyxia, infection, or maternal intake of NSAIDs. The Echo results of right heart enlargement, pulmonary hypertension, and absence of patent ductus arteriosus (PDA) may implicate possible causes of premature closure of ductus arteriosus.

Management of patients with TR caused by rupture of the papillary muscles or chordae tendineae includes surgery and nonsurgical treatment. Surgery is needed to reconstruct the ruptured chordae or papillary muscles in patients presenting right heart circulatory failure. In all 26 reported cases including ours, surgery was performed on 23 cases and surgical intervention was planned on one case. Only one early case in 1995 was reported to die after surgery (15), which might be related to the surgical technique of anterior leaflet excision and valvuloplasty. Two cases without severe cardiac failure were advised to follow up and perform well at the 8 month and 1-year follow-ups, respectively (11, 16). The possible explanation is that the two cases have only mild anterior prolapse caused by the ruptured papillary muscle, normal biventricular structure, and no right ventricular outflow tract (RVOT) stenosis. Surgery was performed on almost all patients during neonatal periods, except in one case on whom surgery was performed at 3 months old due to a sudden right heart failure 2 months after the first nonsurgical treatment (3). The surgical technique is still under debate. Neochordae can be artificial or reconstructed by reimplanting the head of the ruptured papillary muscle into the residual base on the endocardium. The advantages of artificial chordae include being a well-established technique, easy availability, low cost, and ease of use. Mid-term follow-ups have revealed satisfactory results in applying artificial chordae in tricuspid valves, but the disadvantage of lack of growth potential is still a concern. The reimplantation technique also performed well after surgery in six patients, and long-term research was also needed. A proper strategy should be chosen according to the intraoperative findings. Although surgery repair is important, recurrent rupture may occur if pulmonary vascular resistance (PVR) is still high. Nonsurgical treatment includes NO, sildenafil, oxygen supplement, and mechanical ventilation to stabilize hemodynamic performance and lower PVR. In most patients, PVR decreased and the use of extracorporeal membrane oxygenation (ECMO) was avoided after the comprehensive treatment. Previous reports indicated that ECMO was a reliable method for cardiopulmonary support in neonates who required ECMO as a bridge to surgical repair (2, 3).

Long-term follow-up is rarely reported. Chowdhuri et al. (17) reported five cases with a median follow-up of 89 months. All five patients grew normally during follow-up and had either trivial or mild TR with good coaptation of the TV at the last visit. Anagnostopoulos et al. (18) reported a case with postoperative seizures and left frontal lobe infarction but recovered well after treatment. Echo results at 4 years old revealed normal biventricular function and trivial TR.

## Conclusions

To summarize, unguarded severe TR caused by rupture of the papillary muscles or chordae tendineae is rare but potentially fatal in neonates. Prompt diagnosis by Echo, proper comprehensive preoperative management, and timely surgery can be life-saving and improve prognosis.

## Data Availability

The raw data supporting the conclusions of this article will be made available by the authors, without undue reservation.

## References

[1] Gonzalez-Lopez MT Perez-Caballero-Martinez R Gil-Jaurena JM. Myocarditis, flail tricuspid valve, and normal rhythm: an exceptional form of neonatal cardiac lupus. Cardiol Young. (2017) 27(7):1419–22. 10.1017/S1047951117000543 28460654

[2] Yoon JK Kim HR Kwon HW Kwon BS Kim GB Bae EJ Ruptured tricuspid valve papillary muscle in a neonate with intractable persistent fetal circulation. Korean Circ J. (2015) 45(4):340–3. 10.4070/kcj.2015.45.4.340 26240590 PMC4521114

[3] Fleming GA Scholl FG Kavanaugh-McHugh A Liske MR. A case of an infant with flail tricuspid valve due to spontaneous papillary muscle rupture: was neonatal lupus the culprit? Pediatr Cardiol. (2008) 29(2):442–5. 10.1007/s00246-007-9109-8 17882476

[4] Saker DM Walsh-Sukys M Spector M Zahka KG. Cardiac recovery and survival after neonatal myocardial infarction. Pediatr Cardiol. (1997) 18(2):139–42. 10.1007/s002469900133 9049129

[5] Lim KA Huh J Jun TG. Successful repair of critical tricuspid regurgitation secondary to ruptured papillary muscle in a newborn. Cardiol Young. (2004) 14(4):450–2. 10.1017/S1047951104004172 15680055

[6] Katogi T Aeba R Ito T Goto T Cho Y Ueda T Surgical management of isolated congenital tricuspid regurgitation. Ann Thorac Surg. (1998) 66(5):1571–4. 10.1016/S0003-4975(98)00753-X 9875753

[7] Bucciarelli RL Nelson RM Egan EA Eitzman DV Gessner IH. Transient tricuspid insufficiency of the newborn: a form of myocardial dysfunction in stressed newborns. Pediatrics. (1977) 59(3):330–7. 10.1542/peds.59.3.330 138840

[8] Marton T Hajdú J Hruby E Papp Z. Intrauterine left chamber myocardial infarction of the heart and hydrops fetalis in the recipient fetus due to twin-to-twin transfusion syndrome. Prenat Diagn. (2002) 22(3):241–3. 10.1002/pd.302 11920902

[9] Lazda EJ Batchelor WH Cox PM. Immunohistochemical detection of myocardial necrosis in stillbirth and neonatal death. Pediatr Dev Pathol. (2000) 3(1):40–7. 10.1007/s100240050005 10594130

[10] De Busk RF Harrison DC. The clinical spectrum of papillary-muscle disease. N Engl J Med. (1969) 281(26):1458–67. 10.1056/NEJM196912252812607 4901023

[11] Inatomi A Sasahara J Ishii K Nobuaki M. Prenatal diagnosis of premature constriction of the ductus arteriosus with tricuspid papillary muscle rupture: a case report. J Med Ultrason. (2018) 45(2):337–40. 10.1007/s10396-017-0807-4 28725980

[12] Kaulitz R Haen S Sieverding L Ziemer G. Intrauterine rupture of anterior tricuspid valve papillary muscle: tricuspid valve chordae replacement on the first day of life. J Thorac Cardiovasc Surg. (2012) 143(1):241–3. 10.1016/j.jtcvs.2011.07.015 21855897

[13] Sachdeva R Fiser RT Morrow WR Cava JR Ghanayem NS Jaquiss RD Ruptured tricuspid valve papillary muscle: a treatable cause of neonatal cyanosis. Ann Thorac Surg. (2007) 83(2):680–2. 10.1016/j.athoracsur.2006.06.054 17258015

[14] Sari G Oteyaka E Kuguoglu OE Basunlu MT Karakurt Y Yozgat Y Congenital tricuspid insufficiency due to rupture of chordae tendinea secondary to intrauterine obliteration of ductus arteriosus. Cardiol Young. (2022):1–4. 10.1017/S1047951122001482 35545882

[15] Atalay S Imamoglu A Uluoglu O Ikizler C. Critical tricuspid regurgitation secondary to ruptured chordae tendineae mimicking a mass on the tricuspid valve in a newborn. Pediatr Cardiol. (1995) 16(3):133–6. 10.1007/BF00801912 7617508

[16] Yemul MA. A flail anterior tricuspid leaflet secondary to anteroposterior papillary muscle rupture in a neonate. Echocardiography. (2014) 31(5):E151–5. 10.1111/echo.12522 24495104

[17] Roy Chowdhuri K Dutta N Raja N Girotra S Radhakrishnan S Iyer PU Mid-term follow-up of neonatal neochordal reconstruction of tricuspid valve for perinatal chordal rupture causing severe tricuspid valve regurgitation. World J Pediatr Congenit Heart Surg. (2020) 11(5):587–94. 10.1177/2150135120929011 32853064

[18] Anagnostopoulos PV Alphonso N Nolke L Hornberger LK Raff GW Azakie A Neonatal mitral and tricuspid valve repair for in utero papillary muscle rupture. Ann Thorac Surg. (2007) 83(4):1458–62. 10.1016/j.athoracsur.2006.10.077 17383357

